# Effects of Different Pretreatments of DNA Extraction from Dried Specimens of Ladybird Beetles (Coleoptera: Coccinellidae)

**DOI:** 10.3390/insects10040091

**Published:** 2019-03-29

**Authors:** Weidong Huang, Xiufeng Xie, Xinyue Liang, Xingmin Wang, Xiaosheng Chen

**Affiliations:** 1 Guangdong Key Laboratory for Innovative Development and Utilization of Forest Plant Germplasm, Department of Forest Protection, College of Forestry and Landscape Architecture, South China Agricultural University, Guangzhou 510640, China; wdh6434@163.com (W.H.); 18316028116@163.com (X.L.); 2 Key laboratory of Bio-pesticide Innovation and Application, Engineering Technology Research Center of Agricultural Pest Biocontrol, Department of Entomology, South China Agricultural University, Guangzhou 510640, China; wangxmcn@scau.edu.cn; 3 Guangdong Agriculture Industry Business Polytechnic College, Guangzhou 510507, China; xfxie@gdaib.edu.cn

**Keywords:** pretreatment, DNA extraction, dry specimens, phylogenetic analysis, Coccinellidae

## Abstract

Obtaining genetic information from museum specimens is a fundamental component of many fields of research, including DNA barcoding, population genetics, conservation genetics, and phylogenetic analysis. However, acquiring genetic information from museum specimens is challenging because of the difficulty in amplifying the target sequences due to DNA damage and degradation. Different pretreatments can significantly impact the purity and concentration of genomic DNA from museum specimens. Here, we assessed four pretreatment methods—use of 0.9% NaCl buffer, phosphate-buffered saline (PBS), Saline Tris-EDTA (STE) buffer, and sterile water—to determine which pretreatment is most suitable for DNA extraction from dried specimens of ladybird beetles. We completed a comprehensive phylogenetic analysis to test whether the sequences obtained from dried specimens enable proper phylogenetic inference. Our results showed that pretreatment can improve the quality of DNA from dried specimens. The pretreatment effects of 0.9% NaCl buffer and STE buffer were better than those of PBS buffer and sterile water. The phylogenetic analyses results showed that museum specimens can be used to generate cogent phylogenetic inferences. We report the optimum pretreatment methods for DNA extraction from dried ladybird beetles specimens as well as provide evidence for accurately determining phylogenetic relationships for museum specimens.

## 1. Introduction

Developments in molecular biology, specimen identification, and phylogenetic and population genetics require the use of molecular techniques, and DNA sequences provide vast quantities of information for phylogenetic inference and taxonomic identification. However, obtaining and collecting fresh material is time-consuming, expensive, and often fails to provide a wide coverage of the species [1]. Museum specimens generally cover a broader taxonomic range and are more easily obtained, enabling a wider range of questions and taxa to be studied [2]. Many researchers are trying to exploit this potential of museum specimens [3,4,5] and some museums are becoming active molecular genetics research institutions. Museums worldwide house millions of animal and plant specimens, many of which have been preserved so that scientific investigation is possible. Hence, museum specimens have become an important source of data in population genetics, conservation genetics and phylogenetic inference studies [2]. For research on museum specimens, high-quality DNA from dried specimens is required.

Advancements in molecular biology have enabled the extraction of genomic DNA from historic and even ancient tissue specimens [6]. However, evident limitations exist when using DNA from museum specimens; obtaining sufficient amounts of high-quality DNA is the main challenge. In general, not only age but also storage and preservation methods affect DNA quality and the amplification success [1]. Results of earlier studies indicated that different pretreatment methods can significantly impact the purity and concentration of DNA extracts from dried insect specimens [7,8,9,10].

Generally, many museum specimens, particularly dry-preserved insects, are stored pinned without any further preservation treatment [11]. Whereas the exoskeleton of the insects is stable for many years, the soft tissue soon dries out and decomposes [12]. Consequently, pretreatment is extremely important for DNA extraction from dried insect specimens. Previous studies reported a significant impact of pretreatment on the quality and purity of dried insect specimen DNA, favorable for polymerase chain reaction (PCR) success and other molecular techniques [7,8].

Phylogenetic studies are vital for addressing biological questions as about the relationships among species or genes, the origin and spread of species and the demographic changes and migration patterns of species [13]. Therefore, using museum specimens can help with forming correct phylogenetic inferences, also enabling a wider range of questions to be studied. In the present study, we assessed the effects of different kinds of DNA extraction pretreatment from dried specimen. We chose specimens of ladybird beetles (Coleoptera: Coccinellidae) for the current study as the species is highly abundant in the temperate zone, being important insect natural enemies and the subject of many ecological studies [14]. We amplified two mitochondrial genes: cytochrome oxidase subunit I (*COI*) and 16S ribosomal RNA (*16S*) and one nuclear gene histone subunit 3 (*H3*) from dried ladybird beetles, and then applied four dominant phylogenetic analysis methods—neighbor-joining (NJ), maximum parsimony (MP), maximum likelihood (ML), and Bayesian inference (BI)—to test whether these sequences are an appropriate basis for phylogenetic inference. The objectives of this study were to (1) identify a best-practice approach for pretreatment of high-quality DNA extracts from dried specimens, and (2) test whether museum specimens can be used successfully for determining reproducible phylogenetic relationships.

## 2. Materials and Methods

### 2.1. Specimens

All specimens were obtained from the Engineering Research Center of Biological Control, Ministry of Education, South China Agriculture University (SCAU, Guangzhou, China). Dried pinned specimens of *Afissula expansa* (Dieke, 1947), *Epilachna plicata* Weise, 1889, *Hippodamia variegata* (Goeze, 1777) and *Scymnus* (*Pullus*) *kawamurai* (Ohta, 1929) were selected. In total, we collected 20 samples, 5 of each species, and applied the 4 methods of pretreatment and a blank control (Table 1). Samples were checked if they were mildewed and vermiculated under a stereoscope, to ensure there was no cross-contamination of DNA from fungi or something else.

### 2.2. Pretreatments

The specimens each underwent one of the following methods: 0.9% NaCl buffer, Saline Tris-EDTA (STE) buffer (0.1 mol/L NaCl, 10 mmol/L Tris·Cl pH 8.0, 1 mmol/L ethylene diamine tetraacetic acid (EDTA) pH 8.0), phosphate-buffered saline (PBS) buffer, or sterile water. All specimens were placed in a reaction tube. After the respective pretreatment, buffer solution or sterile water was added until the specimens were submerged and steeped for 3 h at room temperature. Likewise, the blank control was placed in a reaction tube and allowed to sit for 3 h at room temperature without any further treatment.

### 2.3. DNA Extraction and PCR Amplification

All samples were moved to a new reaction tube for total genomic DNA extraction following pretreatment. All DNA extractions were completed with the Qiagen DNA Blood and Tissue kit (TianGen Biochemistry, Beijing, China) following the protocol provided by the manufacturer. A spectrophotometer (NanodropTM, ThermoFisher Scientific, Waltham, MA, USA) was used to measure the OD_260/280_ ratio for characterizing the DNA quality. An OD_260/280_ value below 1.6 indicates that the DNA extract has been contaminated by protein or phenol, whereas an OD_260/280_ value above 1.9 indicates that the DNA extract has been contaminated by RNA.

Two mitochondrial genes, cytochrome-c oxidase subunit I (*COI*) and 16S ribosomal RNA (*16S*) and one nuclear gene histone subunit 3 (*H3*), were amplified to assess the quality of the DNA extracts. PCR was conducted in 25 µL volumes including 12 µL SuperMix (TransGen Biotech, Beijing, China), 10 µL deionized H_2_O, 1 µL template and 1 µL each of primer. PCR cycle conditions for the three genomic regions were similar: an initial denaturation of 3 min at 94 °C, followed by 35 cycles of 30 s at 94 °C, 30 s at 50 °C and 1 min at 72 °C, and a final extension at 72 °C for 5 min. PCR products were electrophoresed on 1.0% agarose gel. DNA fragments were sequenced in both directions with sufficient overlap to ensure the accuracy of sequence data. Sequencing was performed by Sangon Biotech, Shanghai, China. Raw sequences were assembled and edited in Geneious 9.1.5 [17], manually checked for sequencing errors and ambiguities, and then BLASTed in GenBank. The detail information is shown in Table 2.

### 2.4. Sequences Composition and Phylogenetic Analysis

The base composition and the number of parsimony informative sites were calculated using MEGA 7.0 [18]. Alignments of the individual makers were linked in SequenceMatrix [19]. Neighbor-Joining (NJ), maximum parsimony (MP), maximum likelihood (ML) and Bayesian inference (BI) approaches were applied to test whether these sequences enable correct phylogenetic inference.

An NJ tree was constructed in MEGA 7.0 based on the Kimura-2-parameter (K2P) model using a combined dataset. *Endomychus biguttatus* Say (*COI*: GQ302304, *16S*: GQ302094, and *H3*: GQ302447) and *Corynomalus vestitus* Voet (*COI*: GQ302321, *16S*: GQ302115, and *H3*: GQ302462) were downloaded from GenBank as an outgroup to root the tree. These species belong to the Endomychidae. This family has been identified as a sister-group of the Coccinellidae within superfamily Coccinelloidea [20,21]. MP analyses were performed using PAUP 4.0 [22]. Heuristic searches were conducted using tree bisection reconnection (TBR) branch swapping, with 1000 random-addition replicates. ML and BI analyses were performed in RAxML 8.2.8 [23] and MRBAYES 3.2.6 [24]. Modeltest [25] was used to select an appropriate model of sequence evolution for each gene under the Akaike information criteria (AIC). PartitionFinder 1.1.1 [26] was used to find the best-fit substitution model for each partition based on the synthesized dataset. The analysis was run using all search schemes, with all models considered based on the AIC. ML was analyzed using the 1000 rapid bootstrapping replicates. For BI analyses, all model parameters were unlinked. Two MCMC runs were conducted with one cold chain and three-headed chains (temperature set to 0.1) for 20 million generations and sampled every 1000 generations. The first 25% of the total trees were discarded as ‘burn-in’ and the remaining trees were used to generate a majority-rule consensus tree. The chain stationarity was visualized by plotting likelihoods against the generation number using the program TRACER 1.6 [27].

## 3. Results

### 3.1. Measurement of DNA Purity and Concentration

DNA purity and concentration varied amongst the different pretreatments (Table 3). The OD_260/280_ value was species specific. The OD_260/280_ for four different pretreatment methods with *E*. *plicata* and *S*. (*P*.) *kawamurai* resulted in higher purity values whereas the OD_260/280_ for *H*. *variegata* indicated that the DNA extracts may have been contaminated by RNA. The different pretreatment methods impacted DNA purity, especially for *E*. *plicata* and *S*. (*P*.) *kawamurai*. However, the OD_260/280_ of the sterile water treatment was 2.003, which was greater than the value for the blank control (1.983) in *A*. *expansa*. Apart from DNA purity, different pretreatments impacted on DNA concentration. Here, the DNA concentration of *E*. *plicata*, *H*. *variegata*, and *S*. (*P*.) *kawamurai* was higher than those of the blank control.

### 3.2. Amplification of Sequences

**Figure 1:**
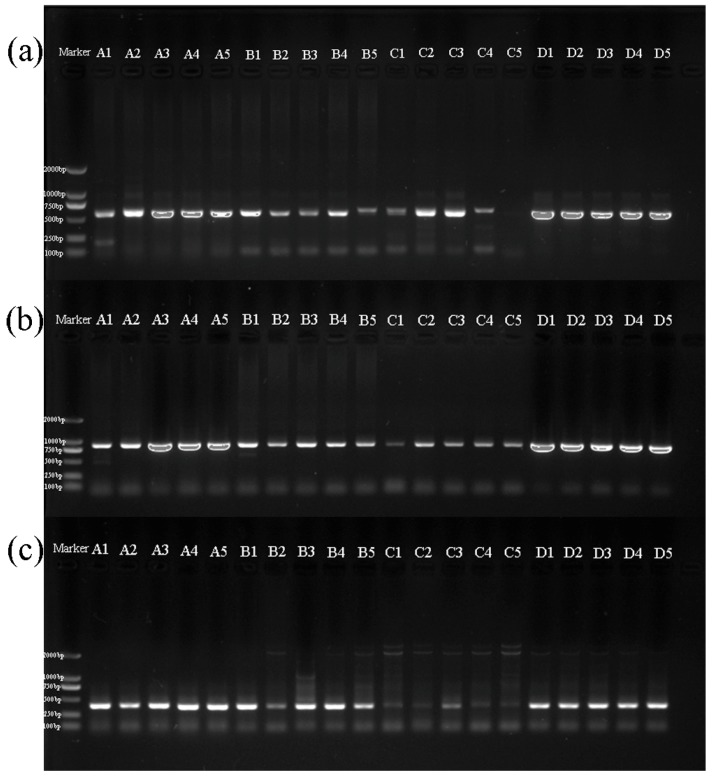
Polymerase chain reaction (PCR) amplification of three genes: (**a**) *16S*, (**b**) *COI*, and (**c**) *H3*. The code in the agarose gel image corresponds to the code in Table 1.

**Figure 2:**
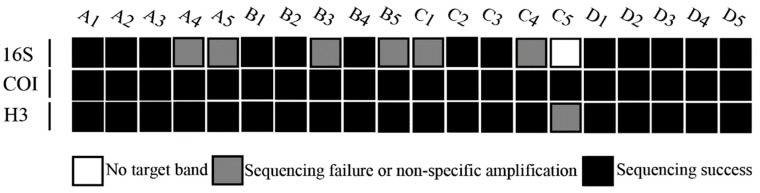
Amplification efficiency of *16S*, *COI* and *H3*. Three different shades of squares are used to represent the PCR and sequencing results. The code in this figure corresponds to the code in Table 1.

In total, we obtained 58 DNA sequences, and all sequences were BLASTed in GenBank. All *COI* and *H3* fragments were confirmed as the targeted genomic region whereas *16S* had six sequences that were non-specifically amplified, A4, A5, B3, B5, C1 and C4, and non-specific sequences were removed. Finally, we obtained 52 targeted DNA sequences: 13 *16S* sequences, 20 *COI* sequences and 19 *H3* sequences. The target bands of *16S*, *COI* and *H3* of *H*. *variegata* on the agarose gel were weak (Figure 1). The blank control of *H*. *variegata* C5 did not produce PCR product for *16S*. Likewise, for *H3* of *H*. *variegata*, the control resulted in no band whereas pretreatment samples displayed an amplified target region (Figure 2).

The edited and aligned sequences lengths for *16S*, *COI*, and *H3* are 523 bp, 867 bp, and 290 bp, respectively. The average contents of A, T, G, and C were 35.7%, 40.7%, 14.9%, and 8.7% for *16S*; 30.6%, 38.1%, 14.7%, and 16.6% for *COI*; and 23.5%, 18.9%, 27.8%, and 29.7% for *H3*, respectively. The number of parsimony informative sites of *16S*, *COI*, and *H3* were 86, 230, and 68, respectively. After linking the three genes, our final aligned fragment length was 1680 bp, which contained 384 parsimony informative sites. The average contents of A, T, G, and C were 30.4%, 35.1%, 17.2%, and 17.3%, respectively.

### 3.3. Phylogenetic Analyses

**Figure 3:**
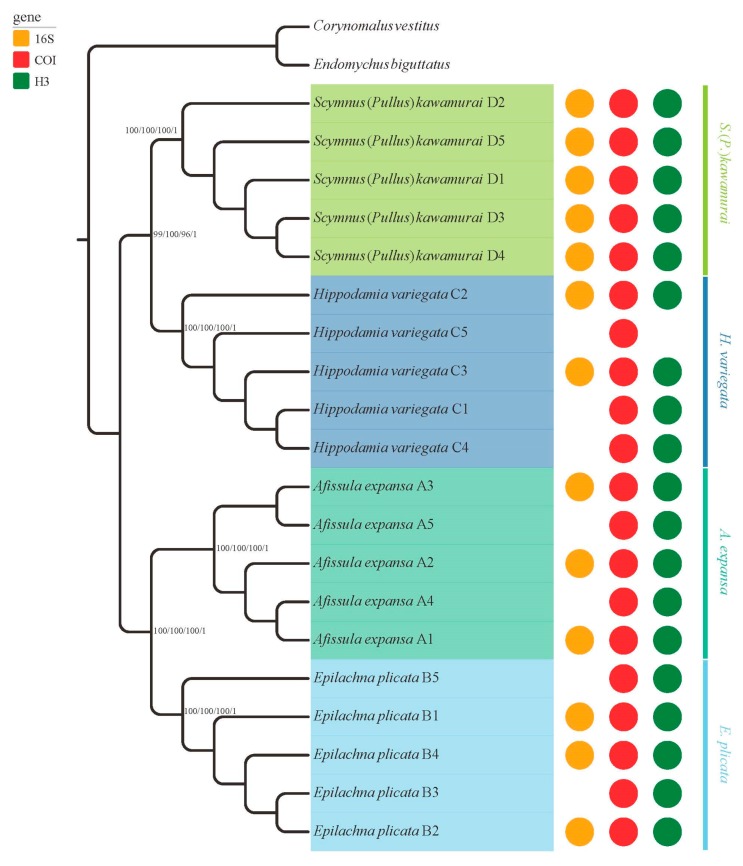
Cladogram derived from analyses of the three markers demonstrating the phylogenetic relationships based on neighbor-joining (NJ), maximum parsimony (MP), maximum likelihood (ML), and Bayesian inference (BI) of the combined dataset (1680 bp) under partition strategies. The support rate of the branches from left to right represents bootstrap values for NJ analysis, bootstrap values for MP analysis, bootstrap values for ML analysis, and posterior probabilities for BI. The codes following the sample name in this figure correspond to the code in Table 1. The vacant part of the circle on the right side of the tree represents target genes that we failed to amplify or sequence.

PartitionFinder results showed that each gene was partitioned separately, and GTR + I + G as the most appropriate model for each gene. Four phylogenetic reconstruction methods (NJ, MP, ML, and BI) of linked data yielded similar topologies (Figure 3). Four main clades could be distinguished, all highly supported statistically. The five samples of the same taxa formed a clade. The AIC, as implemented in Modeltest, yielded the GTR + I + G model of sequence evolution as most appropriate for *COI*, *16S* and *H3*. The phylogenetic results based on each gene were the same as the results based on linked data (Figures S1–S3). Our phylogenetic analyses showed that these sequences obtained from dry specimens could be used for phylogenetic inference.

## 4. Discussion

In the present study, we assessed the effects of four different pretreatment methods on the quality of DNA extracts of museum specimens including the testing of the amplification success using three different genomic regions. Our results indicate that different pretreatments can improve DNA purity and concentration, and the 0.9% NaCl buffer and STE buffer produced the highest DNA purity and quality. Pu et al. [9] stated that genomic DNA could be successfully extracted from dried specimens of Hymenoptera, which were collected in 1980 and 1987 using STE buffer pretreatment [9]. An et al. [7] also used STE buffer as a pretreatment for DNA extraction of dried specimens of the tribe Platyopini (Coleoptera: Tenebrionidae), which were collected from 1987 to 2008. Their results showed that high-quality genomic DNA could be extracted and they successfully amplified DNA fragments in PCR. Li et al. [8] compared the pretreatment effects of 0.9% NaCl buffer and sterile water for DNA extraction of *Prostephanus truncatus*, *Callosobruchus maculatus*, and *Sitophilus oryzae* using different reaction times. Their results showed that bathing in 0.9% NaCl buffer for three hours was the best for DNA quality. In this study, the effects of pretreatments by PBS buffer was inferior to 0.9% NaCl buffer and STE buffer in *A. expansa*, *E. plicata* and *S.* (*P.*) *kawamurai*, but not for *H. variegata*. Pretreatments employing PBS buffer have already been shown to increase the DNA quality of specimens stored in alcohol. Generally, PBS gradually restores cells to the original physiological status due to slow permeation and gradually promotion of the cross-linking protein separation of DNA [10]. Conversely, pretreatment with sterile water can lead to severe cell damage due to high water absorption, followed by a dispersion of the cellular contents. Subsequently, partial genomic DNA will be lost [8].

For *COI*, we achieved excellent amplification success, even though this is the longest sequence of the three molecular markers investigated. The nuclear gene *H3* presented 19 target bands, whereas the mitochondrial *16S* had a high PCR success rate, but six sequences presented non-specific amplification. A similar situation was observed for the PCR amplification of DNA from ancient samples [28]. Although mitochondrial DNA is more easily amplified from suboptimal DNA extracts than nuclear genes [29], damaged templates may cause incorrect bases to be incorporated in the PCR product [30]. Most errors involved C→T substitutions on the L-strand, presumably due to deamination of cytosine in the template [31]. For *16S*, PCR and sequencing results indicated that the pretreatments seem to have a positive effect on the quality of the DNA extract in increasing the chance for amplification success, but this was not true for *COI* and *H3*.

Our phylogenetic analyses revealed four highly supported clades, and the same taxa clustered together (Figure 3). Within high-level phylogenetic analyses, both *A. expansa* and *E. plicata* were shown to belong to the tribe Epilachnini [32]. In our analyses, both *A. expansa* and *E. plicata* recovered a monophyly with very good support for their taxonomic status. As mentioned above, our analyses proved that museum specimens can be employed for cogent phylogenetic inference.

## 5. Conclusions

In this study, we assessed the effects of four pretreatment methods—bathing in 0.9% NaCl buffer, PBS buffer, STE buffer, and sterile water—to identify the best practice for high-quality DNA extractions using dried ladybird beetles specimens. Our results showed that pretreatment can improve the quality of DNA. The addition of 0.9% NaCl buffer and STE buffer had better effects than PBS buffer and sterile water. Comprehensive phylogenetic analyses showed that museum specimens can be accurately used for phylogenetic inference. Overall, we identified appropriate pretreatment methods for DNA extraction from dried specimens and provided evidence that museum specimens can be used to correctly determine phylogenetic relationships. In order to effectively identify the most appropriate pretreatment, more replicates per species and treatments are needed in our further studies.

## Figures and Tables

**Table 1:** Information on sample pretreatments, collection date, species and code information of museum specimen of five different coccinellid species used in the experiment.

Pretreatment	Code	Collection Date	Species	GenBank Accession		
				*16S*	*COI*	*H3*
0.9% NaCl buffer	A1	2009	*Afissula expansa*	MK138697	MK190427	MK190447
PBS buffer	A2	2009	*A. expansa*	MK138698	MK190428	MK190448
STE buffer	A3	2009	*A. expansa*	MK138699	MK190429	MK190449
Sterile water	A4	2009	*A. expansa*	-	MK190430	MK190450
CK	A5	2009	*A. expansa*	-	MK190431	MK190451
0.9% NaCl buffer	B1	2009	*Epilachna plicata*	MK138700	MK190432	MK190452
PBS buffer	B2	2009	*E. plicata*	MK138701	MK190433	MK190453
STE buffer	B3	2009	*E. plicata*	-	MK190434	MK190454
Sterile water	B4	2009	*E. plicata*	MK138702	MK190435	MK190455
CK	B5	2009	*E. plicata*	-	MK190436	MK190456
0.9% NaCl buffer	C1	2008	*Hippodamia variegata*	-	MK190437	MK190457
PBS buffer	C2	2008	*H. variegata*	MK138703	MK190438	MK190458
STE buffer	C3	008	*H. variegata*	MK138704	MK190439	MK190459
Sterile water	C4	2008	*H. variegata*	-	MK190440	MK190460
CK	C5	2008	*H. variegata*	-	MK190441	-
0.9% NaCl buffer	D1	2012	*Scymnus* (*Pullus*) *kawamurai*	MK138705	MK190442	MK190461
PBS buffer	D2	2012	*S.* (*P.*) *kawamurai*	MK138706	MK190443	MK190462
STE buffer	D3	2012	*S.* (*P.*) *kawamurai*	MK138707	MK190444	MK190463
Sterile water	D4	2012	*S.* (*P.*) *kawamurai*	MK138708	MK190445	MK190464
CK	D5	2012	*S.* (*P.*) *kawamurai*	MK138709	MK190446	MK190465

**Table 2:** Information on the primer sequences and corresponding genes information.

Marker	Primer Name	Primer Sequence (5′-3′)	Reference
*COI*	Jerry	CAACATTTATTTTGATTTTTT	[15]
	Spat	GCACTAWTCTGCCATATTAGA	[15]
*16S*	16S A	CGCCTGTTTATCAAAAACAT	[16]
	16S B	CTCCGGTTTGAACTCAGATCA	[16]
*H3*	H3F-1	CAGAAAGTCGACCGGAGGCAAG	This study
	H3R-1	GCGTTTCGCGTGAATGGCG	This study

**Table 3:** DNA purity and concentration of the different pretreatments. The code in this table corresponds to the code in Table 1.

Pretreatment	Code	OD_260/280_	Concentration (ng/µL)
0.9% NaCl buffer	A1	1.936	433.159
	B1	1.798	44.513
	C1	2.075	61.52
	D1	1.899	69.99
PBS buffer	A2	1.966	539.647
	B2	1.929	202.086
	C2	2.067	34.331
	D2	1.924	20.108
STE buffer	A3	1.942	464.019
	B3	1.823	93.246
	C3	2.074	31.527
	D3	1.864	26.774
Sterile water	A4	2.003	678.726
	B4	1.98	52.972
	C4	2.071	32.554
	D4	1.947	17.117
CK	A5	1.983	537.078
	B5	2.007	26.408
	C5	2.098	14.513
	D5	2.090	23.265

## Supplementary Materials

The following are available online at https://www.mdpi.com/2075-4450/10/4/91/s1, Figure S1: Cladogram derived from analyses of the *COI* markers; Figure S2: Cladogram derived from analyses of the *16S* markers; Figure S3: Cladogram derived from analyses of the *H3* markers.
