# Supplementary figures and images for: Effects of Different Pretreatments of DNA Extraction from Dried Specimens of Ladybird Beetles (Coleoptera: Coccinellidae)
Source: Insects. 2019 Mar 29;10(4):91. doi: 10.3390/insects10040091 (PMC6523959; doi:10.3390/insects10040091)

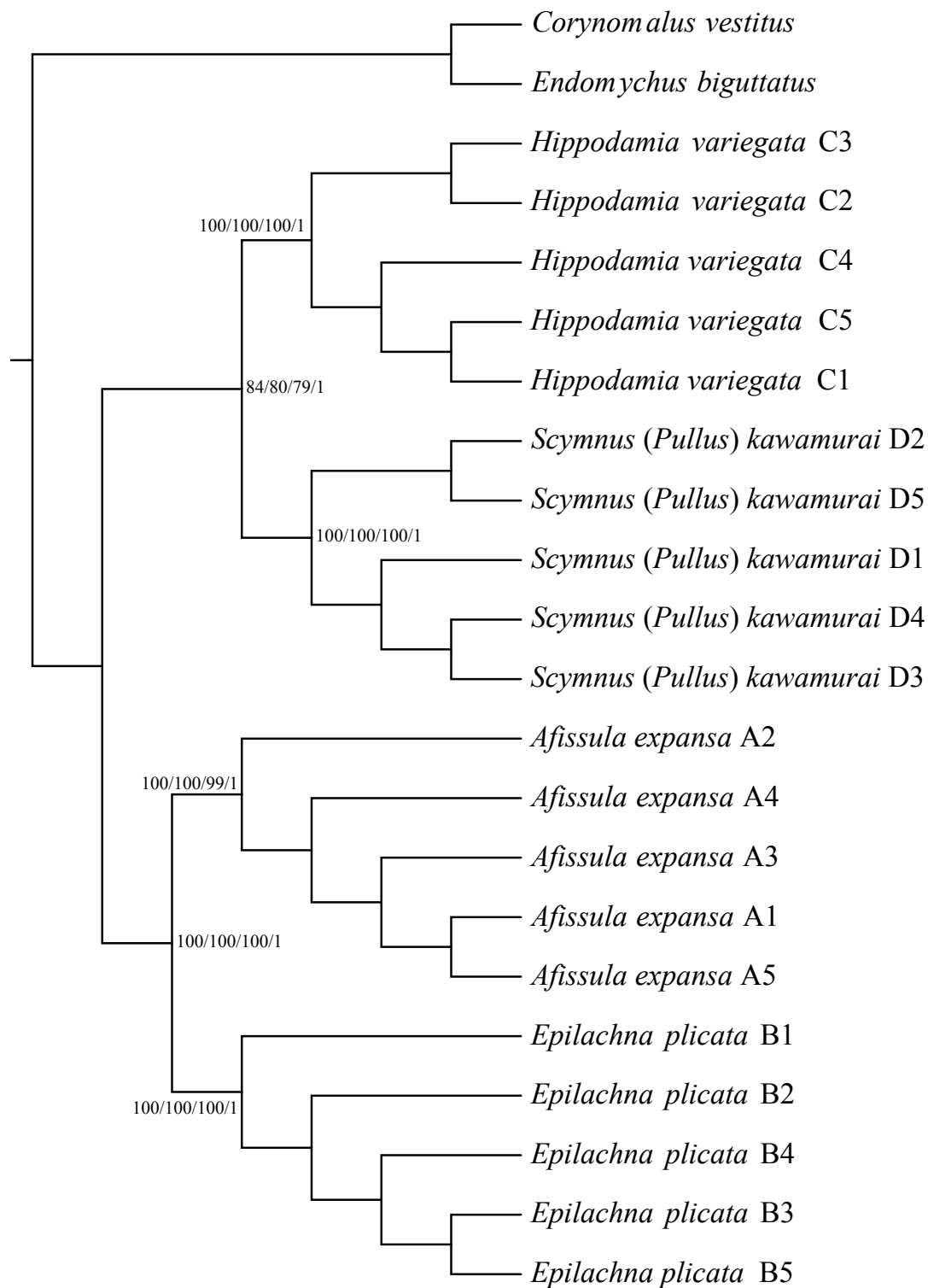

Supplement: Supplementary file 1 [file insects-10-00091-s001.zip › Figure S1.Cladogram derived from analyses of the COI markers.pdf]

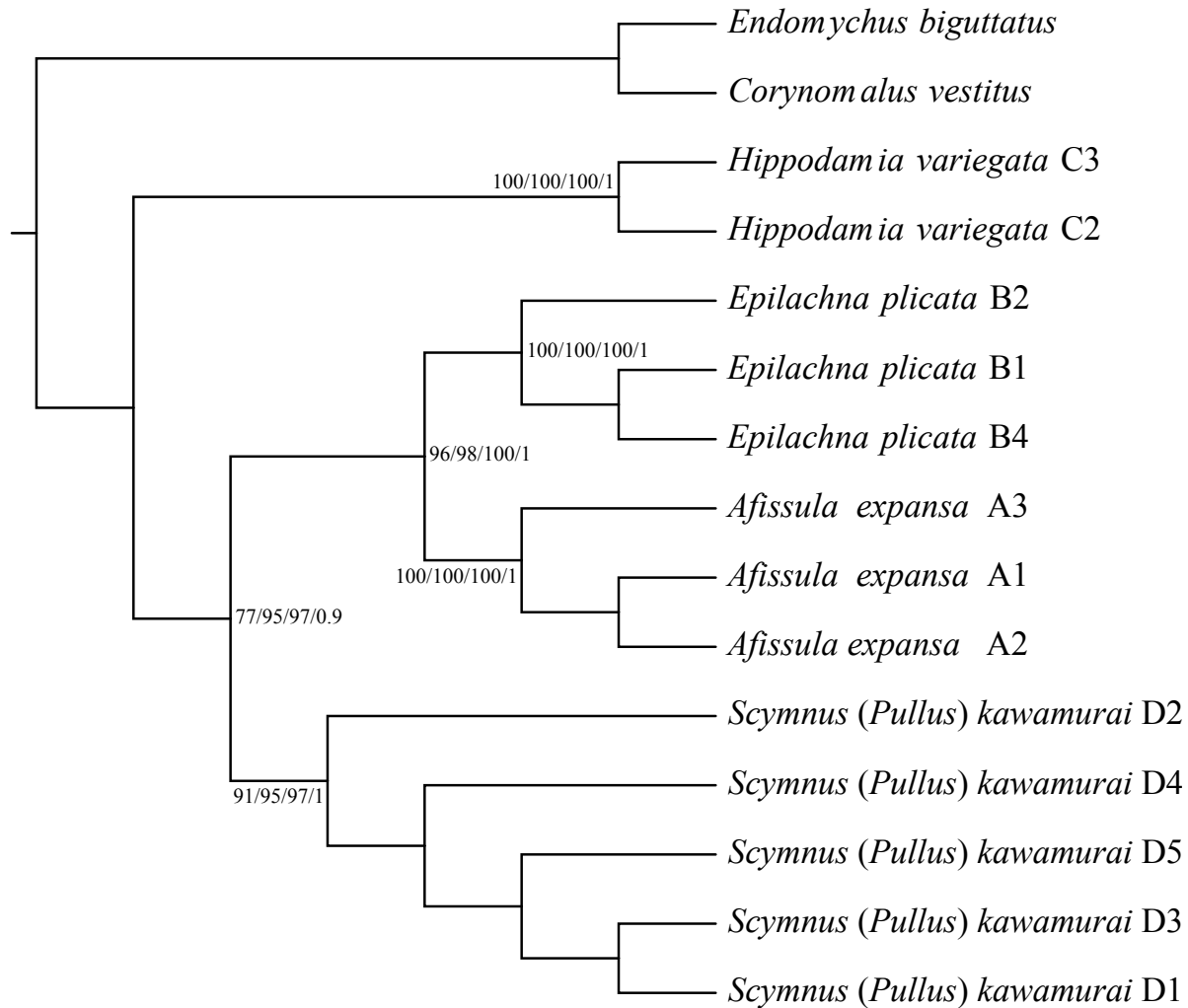

Supplement: Supplementary file 1 [file insects-10-00091-s001.zip › Figure S2.Cladogram derived from analyses of the 16S markers.pdf]

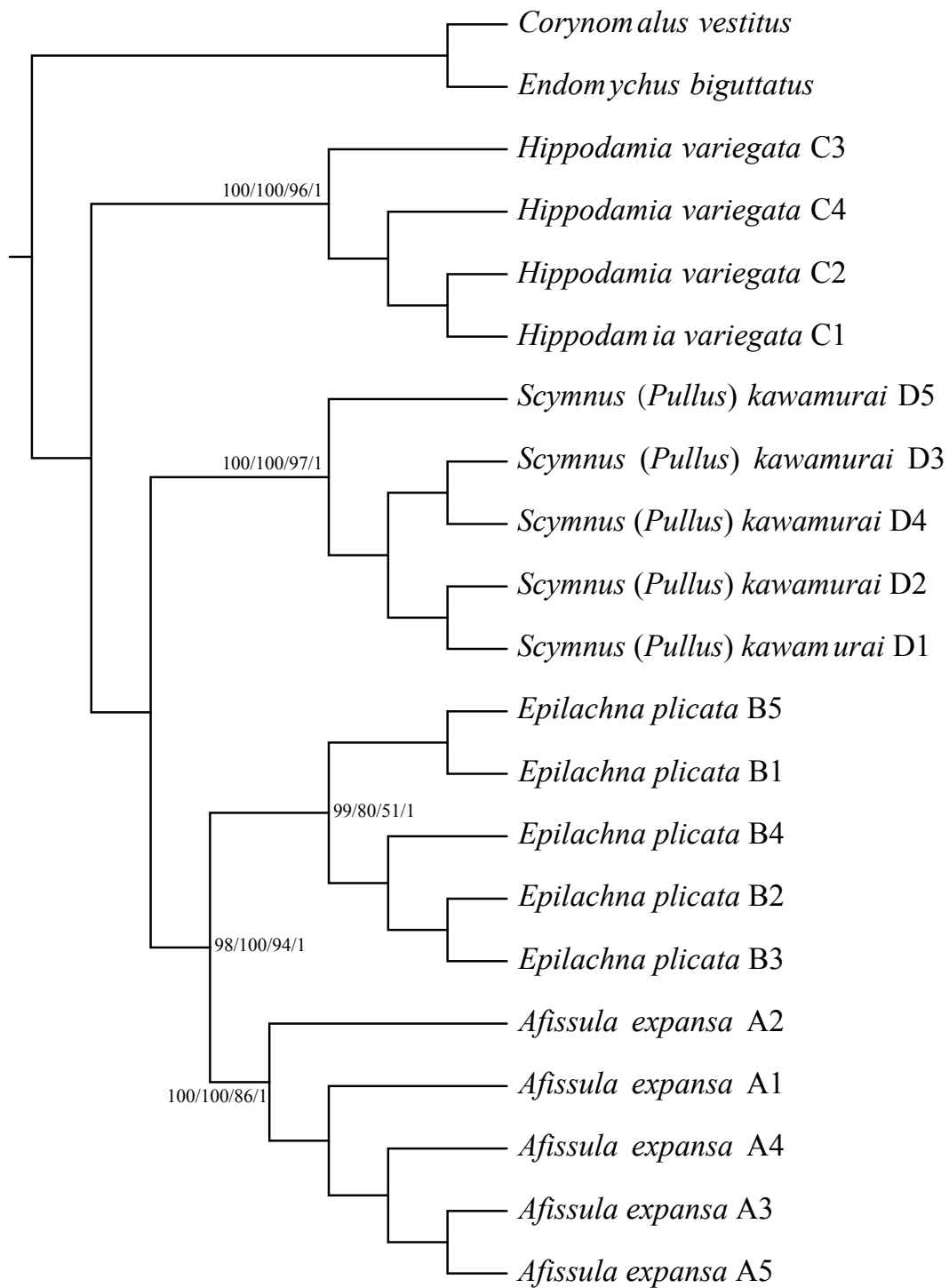

Supplement: Supplementary file 1 [file insects-10-00091-s001.zip › Figure S3.Cladogram derived from analyses of the H3 markers.pdf]
